# Copper Iodide Interlayer for Improved Charge Extraction and Stability of Inverted Perovskite Solar Cells

**DOI:** 10.3390/ma12091406

**Published:** 2019-04-30

**Authors:** Danila Saranin, Pavel Gostischev, Dmitry Tatarinov, Inga Ermanova, Vsevolod Mazov, Dmitry Muratov, Alexey Tameev, Denis Kuznetsov, Sergey Didenko, Aldo Di Carlo

**Affiliations:** 1 L.A.S.E.—Laboratory for Advanced Solar Energy, National University of Science and Technology “MISiS”, Leninskiy prospect 6, Moscow 119049, Russia; saranin.ds@misis.ru (D.S.); gostischev.pa@misis.ru (P.G.); tatarinov_dmitry1994@mail.ru (D.T.); ermanova.io@misis.ru (I.E.); mazov.v@misis.ru (V.M.); muratov@misis.ru (D.M.); 2 Laboratory “Electronic and photon processes in polymer nanomaterials”, Russian Academy of Sciences A.N. Frumkin Institute of Physical chemistry and Electrochemistry, Leninskiy prospect 31k4, Moscow 119071, Russia; a.tameev@gmail.com; 3 Department of Functional Nano Systems and High-Temperature Materials, National University of Science and Technology “MISiS”, Leninskiy prospect 4, Moscow 119049, Russia; dk@misis.ru; 4 Department of Semiconductor Electronics and Device Physics, National University of Science and Technology “MISiS”, Krymskiy val 3, Moscow 119049, Russia; sdi13@mail.ru; 5 CHOSE—Centre for Hybrid and Organic Solar Energy, Department of Electronic Engineering, University of Rome Tor Vergata, via del Politecnico 1, 00133 Rome, Italy

**Keywords:** Inverted perovskite solar cells, passivation, interface stability

## Abstract

Nickel oxide (NiO) is one of the most promising and high-performing Hole Transporting Layer (HTL) in inverted perovskite solar cells due to ideal band alignment with perovskite absorber, wide band gap, and high mobility of charges. At the same time, however, NiO does not provide good contact and trap-free junction for hole collection. In this paper, we examine this problem by developing a double hole transport configuration with a copper iodide (CuI) interlayer for efficient surface passivation. Transient photo-current (TPC) measurements showed that Perovskite/HTL interface with CuI interlayer has an improved hole injection; CuI passivation reduces the concentration of traps and the parasitic charge accumulation that limits the flow of charges. Moreover, we found that CuI protect the HTL/perovskite interface from degradation and consequently improve the stability of the cell. The presence of CuI interlayer induces an improvement of open-circuit voltage V_OC_ (from 1.02 V to 1.07 V), an increase of the shunt resistance R_SH_ (100%), a reduction of the series resistance R_S_ (−30%), and finally a +10% improvement of the solar cell efficiency.

## 1. Introduction

Perovskite solar cells (PSCs) are a breakthrough technology for photovoltaics (PV) that has shown rapid progress in performance achieving 23.7% [1] of power conversion efficiency (PCE) in less than ten years. Such a tremendous development is related to unique semiconductor properties of hybrid halide perovskites owing to long charge carrier diffusion length [2]; damped bulk and surface recombination [3,4]; ambipolar charge transport [5]; and strong absorption in the visible solar spectrum [6]. Moreover, perovskite PV is a solution processing thin film technology that can be realized with low capital expenditure (CAPEX) on rigid as well as on flexible substrates via roll-to-roll printing [7]. At the same time, however, PSCs have not reached the maturity for real industrial exploitation mainly due to issues with device stability [8]. Moreover, hysteresis effects in the current-voltage (JV) characteristics, originating by ion motion and recombination effects at the interfaces [9,10,11] penalize the maximum power point extracted form PSCs. JV characteristic hysteresis can be reduced with the use of inverted planar p-i-n structure without a significant loss in performance [12] and with improved stability under light exposure (with UV-A part) [13,14,15]. The hole transporting layer (HTL) in inverted planar PSC should be chosen considering: (i) the valence band level alignment with respect to perovskite absorber (around −5.45 eV), (ii) a wide band gap to reduce parasitic absorption and enhance electron blocking properties (≥3.0 eV), and (iii) suitable hole mobility range close to halide perovskite 10^−3^–10^1^ V·s/cm^2^). Moreover, HTL fabrication should satisfy some technological requests such as uniformity and planarity of the deposited HTL and, preferably, low-temperature processing. Several organic and inorganic hole transport materials (HTM) were developed for this task and one of the most promising candidates is nickel oxide, which satisfies all the key points listed above. Nickel oxide (NiO) has a 3.6 eV band gap [16], a valence band level E_V_ between −5.4 eV and −5.2 eV, and hole mobilities (µ_h_) up to 10^1^ cm^2^/V·s [17,18]. 

In comparison to organic HTMs, NiO has several advantages such as the good interface stability with current collecting electrodes (opposite to poly(3,4-ethylenedioxythiophene)-poly(styrenesulfonate) (PEDOT:PSS)) that interacts with electrode metal due to its acidity [19]) and a simpler, inexpensive synthesis route with respect to high performing HTLs such as Spiro-OMeTAD and poly[bis(4-phenyl)(2,4,6-trimethylphenyl)amine] (PTAA) [20]. NiO can be deposited with various techniques: solution processing with the use of precursors [21,22]; nanoparticles dispersions [23]; vacuum processing with magnetron sputtering (MS) [24], and atomic layer deposition (ALD) [25]. So far, p-i-n PSCs with NiO HTM demonstrated a Power Conversion Efficiency (PCE) exceeding 18% [26] with a still large room for optimization. However, the intrinsic and not passivated NiO film does not provide a stable junction with perovskite requiring a proper interface modification. Kim et al. [27] showed that the solution processed NiO film contains separated Ni phase that increases interface recombination and reduces the charge transport properties. To solve this problem, the authors proposed a Cs-doped NiO that improved carrier injection and consequently PCE. 

Similarly, several other report focused on doping NiO with Cu, Mg, and Li [28,29,30]. Recently, Jo et al. [31] demonstrated a relative improvement in PCE (+25 %) and charge extraction considering NiO surface passivation with conjugated polyelectrolyte (PhNa-1T) in a double HTL configuration. PhNa-1T allows for the formation of a smooth surface with a decrease of pinholes density and an improved perovskite crystallinity without losing in the stability of the device. A relatively similar combination of effects was presented in the work of Wang et el. [32], where self-assembled monolayers (SAMs) of benzoic acid with positive dipole moments were used as an interfacial film between the HTL and the absorber. SAM modification improves films contact, minimizes energy level offsets, and enhances perovskite crystallization, increasing PSC’s performance with PCE of 18.4% for NiO/SAM HTL much larger than the one obtained for the bare NiO HTL (15.5%). Similarly, Xie et al. [33] used ferrocenedicarboxylic acid to modify NiO surface and obtained improvement in UV durability with the enhancement of PCE from 15.13% to 18.20%. In summary, the NiO–perovskite interface can be improved by doping the NiO or passivating its surface with various types of materials such as small organic molecules, electrolytes, SAMs, and acids. However, pure inorganic surface modifier analogous with MgO for electronic transport layer has not yet been reported [34]. 

**Figure 1:**
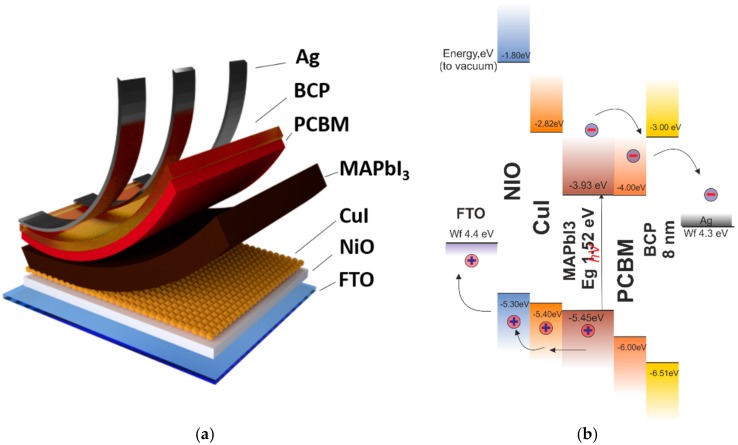
(**a**) Device schematics for inverted perovskite solar cells (PSC) with copper (iodide) CuI passivation layer; (**b**) band profile for PSC with nickel oxide (NiO)/CuI Hole Transporting Layer (HTL) studied in this work (energy levels from [66,67,68]). PCBM: Phenyl-C61-butyric acid methyl ester; (MAPbI_3_): methylammonium lead iodide; BCP: bathocuproine.

In this paper, we demonstrate that commercially available copper iodide can act as efficient passivation material for NiO hole collecting junctions in inverted PSCs. The developed copper iodide (CuI) interlayer was incorporated into the device structure between the perovskite absorber and the NiO transporting layer as shown in Figure 1a. To date, copper iodide was considered as HTL for inverted PSC [35] with max PCE lower than the one achieved with NiO. In this work, however, we show that the main advantage in the use of CuI is in combination with NiO. As presented in the literature, CuI interlayer allows fast hole injection due to its own good transport properties such as high hole mobility up to >40 cm^2^/(V·s) [36,37,38], large lifetime of charges, and high open circuit voltage (V_OC_) when used in heterostructured devices [39,40]. We found that solution processed CuI in the double NiO/CuI HTL configuration can reduce the level of surface recombination improving charge extraction properties and reducing degradation dynamics.

Moreover, the perfect valence band level alignment between Methylammonium Lead Iodide (MAPI) perovskite–CuI–NiO (see Figure 1b) provides no barrier for charge transport. CuI passivation improves the V_OC_ that increases from 1.02 V of the bare NiO HTL up to 1.07 V for NiO/CuI HTL and reduces the series resistance. Finally, a best PCE = 15.26% was shown for the NiO/CuI HTL, corresponding to an increase of almost +10% with respect PSCs with NiO HTL, with an improved shelf life and light soaking stability.

## 2. Materials and Methods 

### 2.1. Materials

All organic solvents—dimethylformamide (DMF), dimethyl sulfoxide (DMSO), chlorobenzene (CB), isopropyl alcohol (IPA), and acetonitrile (ACN)—were purchased in anhydrous, ultra-pure grade from Sigma Aldrich (Munich, Germany), and used as received. Solar cells were fabricated on SnO_2_:F (FTO) coated glass TEC8 from GreatCellSolar (Queanbeyan, Australia). The precursor for NiO hole transporting layer—Tris (ethylenediamine) nickel acetate (TED-NiA)—was synthesized accordingly to the route described in previous work and dissolved in 1.0 M concentration in ethylene glycol (analytical grade). Copper iodide (99.999% trace metal basis, powder) was purchased from Sigma Aldrich. Lead Iodide (99.99%, trace metals basis from TCI Chemicals, Zwijndrecht, Belgium) and methylammonium iodide (MAI, 99.99% purity from GreatcellSolar) were used for perovskite ink. Phenyl-C61-butyric acid methyl ester (PCBM, >99% purity) and bathocuproine (BCP, >99.8% sublimed grade) were purchased from Osilla Inc. (Sheffield, UK) and used for electron transport layers (ETLs) fabrication. 

### 2.2. Inks Preparation

Copper iodide solution was prepared in ACN in several concentrations (0.05 M; 0.10 M and 0.20 M) with vigorous stirring for 8 h. Perovskite ink was made from MAI and PbI2 (1:1 by molar ratio) dissolved in DMF: DMSO mixture (9:1 ratio by volume) at 1.45 M concertation with heating at 60 °C for 6 h. The solution was cooled during five minutes to room temperature before the deposition and filtered through the 0.45 µm polytetrafluoroethylene (PTFE) membrane.

PCBM powder was dissolved in CB (20 mg/mL) and shacked for 90 min before use. BCP was dissolved in IPA (0.5 mg/mL) stirred and heated for at 8 h at 50 °C. All solutions were filtered through 0.45 µm PTFE filter before use.

### 2.3. Device Fabrication

Perovskite solar cell were fabricated with inverted planar architecture FTO/HTL (NiO or NiO/CuI)/ methylammonium lead iodide (MAPbI_3_)/PCBM/BCP/Ag. Firstly, the patterned FTO substrates were cleaned with detergent, de-ionized water, acetone, and IPA in the ultrasonic bath. Then, substrates were activated under UV-ozone irradiation for 30 min. TED–NiA precursor for NiO HTL film was spin coated at 3000 RPMs (60 s), dried at 250 °C (2 min), and annealed at 300 °C (1 h) in the ambient atmosphere. Copper iodide interlayer was spin coated at 3000 RPMs (1 min) and annealed at 100 °C (20 min). MAPbI_3_ absorber film was crystallized on the top of HTL with solvent engineering method. 

Perovskite precursor was spin coated at 4000 RPMs (30 s) and 300 µL of CB were dropped on the substrate on the seventh second after the start of the rotation process. Then, substrates were annealed at 100 °C (10 min) for conversation into black perovskite phase. PCBM solution was spin coated at 1500 RPMs (1 min) and annealed at 50 °C (5 min). BCP interlayer was also spin coated at 4000 RPMs (1 min) and annealed at 50 °C (5 min). Silver cathode was deposited with thermal evaporation method at 2 × 10^−6^ Torr vacuum level trough shadow mask to form 0.15 cm^2^ active area for the pixels of the devices.

Single film thicknesses were measured by stylus profilometer. Firstly, films were deposited on polished Si substrates, then 100 nm of silver was precisely deposited by thermal evaporation on the top to provide uniform solid surface coating. Finally, actual thickness was calculated with subtraction of the scratch step height between the Si substrate level and deposited stack level. For each characterization we measured thickness in five places over the substrate and calculated values with standard deviation. Results presented in Table S1 in supplementary.

### 2.4. Methods of Characterization

Scanning electron microscopy (SEM) images were done with a JEOL JSM7600F system (Tokyo, Japan). Photoluminescence measurements were performed on Cary Eclipse Fluorescence Spectrophotometer with an excitation wavelength of 550 nm. Absorption spectra were measured on Spectronic Helios Alpha UV-Vis spectrophotometer from Thermo Fisher Scientific (Waltham, MA, USA). JV curves were measured under standard conditions of 1.5 AM G illumination and 100 mW cm^−2^ of irradiance intensity using a Newport ABB solar simulator (calibrated with certified Si cell and Ophir irradiance meter). Scan sweeps were performed with Keithley 2400 SMU (Cleveland, OH, USA). A voltage step of 23.5 mV was used for each sweep with a settling time of 10^−2^ s. External quantum efficiency spectra were measured with a QEX10 system from PV measurements (calibration by Si certified cell). Transient photovoltage and photocurrent measurements were performed with Arkeo-Ariadne characterization system (Cicci Research Inc., Grosseto, Italy) with the use of the white LED (CREE XP-G3) for the light source at 1 Sun equivalent intensity. The method for transient measurements consisted of two modes: RISE and FALL. The RISE mode was performed following sharp light illumination from initial dark conditions with 10^−6^ second-time scale resolution for the measurements. The FALL mode was performed in the opposite sequence of transient measurements from illumination to darkness. Maximum power point tracking (MPPT) was done with preliminary forward and reverse scans in the same conditions as for JV measurements at standard 1.5 AM G conditions. Shelf life testing was done accordingly to ISOS-D-1 procedure in the ambient atmosphere.

## 3. Results and Discussions

**Figure 2:**
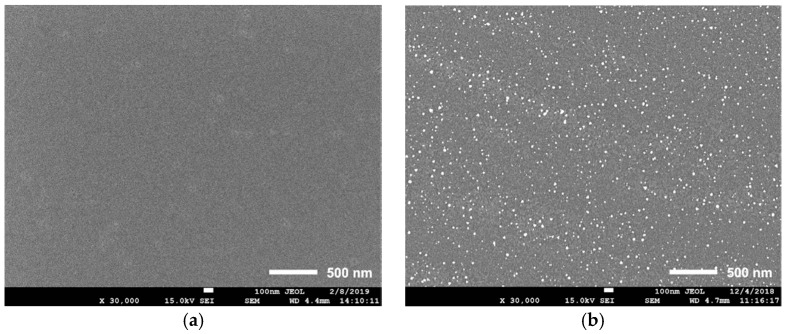

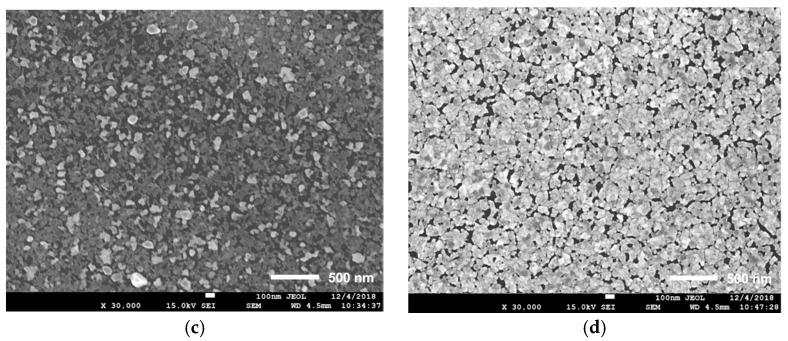
Scanning electron microscopy (SEM) images of CuI coating on the top of NiO concerning concentrations used for the deposition (**a**) reference NiO, (**b**) 0.05 M; (**c**) 0.10 M; and (**d**) 0.20 M.

Firstly, we performed morphology studies for the developed hole transport layers with CuI passivation and reference NiO film. Different HTLs obtained depositing CuI on the top of NiO have been fabricated considering several concentrations of CuI (0.05 M; 0.10 M; 0.20 M) in acetonitrile. Both NiO [41] and CuI were deposited via spin coating (see the experimental section and the device architecture presented in Figure 1). Scanning electron microscopy (SEM) images of CuI film on NiO are reported in Figure 2 (from a to d). Figure 2a shows the smooth amorphous surface of the reference NiO layer with negligible fluctuations in roughness. The presence of copper iodide coating is evident even with the use of the lowest solution concentration (0.05 M) and manifests in separately distributed crystallites (<50 nm size) with web-like areas of coverage showed on Figure 2b. The film continuity improves by increasing the concentration of CuI as it can be seen in Figure 2b–d. We noticed an increased density of CuI nanocrystals for the 0.10 M concentration concerning lower concentrations with sizes up to 100 nm and more extended areas of thin capping film. For a 0.20 M concentration, the CuI layer shows the appearance of long clusters with nanoparticles of different sizes (from tens to hundreds of nanometers). The obtained CuI film morphology compare well with other works where a spin-coated CuI HTL with sub-micron crystallinity was used to fabricate inverted PSCs [42,43]. 

**Figure 3:**
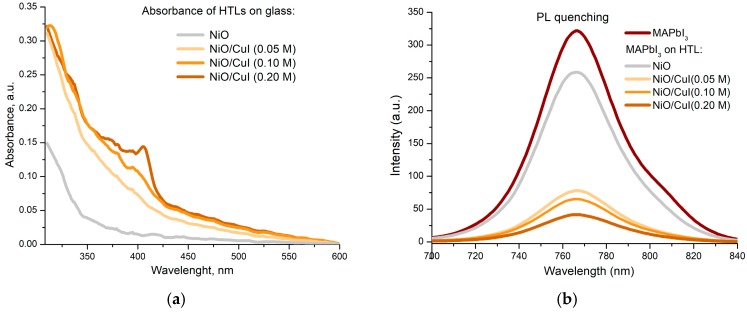
(**a**) The absorbance spectrum of single NiO HTL and double NiO/CuI films; (**b**) MAPbI_3_ photoluminescence (PL) quenching on different HTL configurations.

The absorbance spectrum of the NiO/CuI HTLs for the different concentrations of CuI has been measured to estimate possible optical parasitic losses of the CuI layer. This measurement is an essential point considering that in the inverted PSC the incoming light firstly goes through HTL and then in the perovskite absorber. Figure 3a compares the reference absorption spectra of NiO film on glass with those of the different NiO/CuI HTLs. Even at the smaller concentration, the presence of CuI in the HTL induces an absorption larger than the one of NiO. By increasing the CuI up to 0.2 M, a clear appearance of the CuI band gap absorption around 420 nm is found [44]. Moreover, a residual absorption below the band gap (up to 600 nm) was observed for all NiO-CuI structures. This effect can be related to the Mie resonance effect [45] that occurs in copper iodide nanocrystallites similarly to what we observed for microstuctured semiconductors [46,47,48]. The obtained absorption spectra clearly show that the use of the double HTL increases the optical losses reducing the transparency of the HTL. However, band alignment of the CuI (see Figure 1b) suggests that electron and holes formed after light absorption in CuI can still be efficiently transferred to perovskite and NiO, respectively. Thus, the impact of CuI contribution to the performance of the PSC requires detailed spectral characteristics of the solar cell, which will be presented below. We performed SEM imaging of perovskite films crystalized on NiO and NiO/CuI stack with 0.10 M and 0.20 M concertation of CuI (Figure S1). Pin-hole free perovskite films with ~200–450 nm grain size was obtained without meaningful difference in morphology quality changes. 

To define injection properties of photo-excited charge carriers from the perovskite absorber to HTLs, we analyzed photoluminescence spectra of the single MAPbI_3_ film and changes in the photoemission for multilayer structure-perovskite on NiO and NiO/CuI. The photoluminescence (PL) emission peak for the MAPbI_3_ film (used as a reference on glass) and for HTL/ MAPbI_3_ is shown in Figure 3b for the different single and double HTLs. By introducing the HTL, the MAPbI_3_ PL emission is quenched [41] with respect to pristine MAPbI_3_. This effect is more pronounced in the presence of NiO/CuI HTL with comparison to single NiO HTL. Moreover, by increasing the concentration of CuI, the PL quenching increases. Typically, a strong PL quenching is a signature of enhanced charge transfer from MAPbI_3_ to HTL, which in turn reduces the radiative recombination between electron and holes in the perovskite absorber (PL quenching) and consequently increases charge carrier lifetimes, as reported for polymeric [49] and small molecules [50] transport layers.

**Figure 4:**
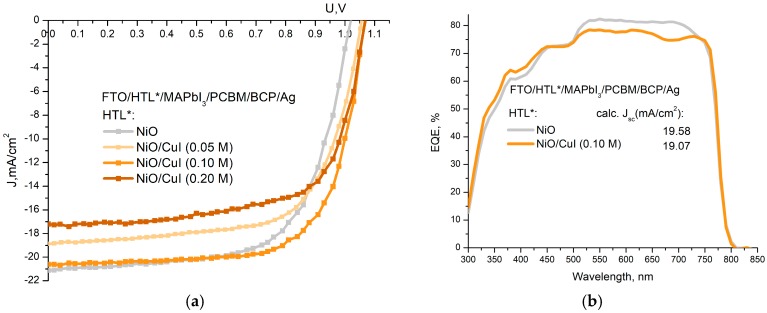
(**a**) Current voltage (JV) curves for a fabricated solar cell with a single NiO HTL and double NiO -CuI HTL, (**b**) external quantum efficiency (EQE) spectra of devices with reference NiO HTL, and best performing CuI interlayer deposited from 0.10 M solution.

Solar cells were fabricated in the inverted planar configuration FTO/HTL/MAPbI_3_/PCBM/BCP/Ag, considering single NiO, CuI, or double NiO/CuI HTLs. The statistical distribution of photovoltaic parameters obtained for the different set of a cell fabricated by varying the HTL is reported in Figure S2 (see E.S.I.). JV characteristics of the best performing PSCs, measured under standard conditions 1.5 AM G (100 mW/cm^2^, 1 sun), are reported in Figure 4a and in Table 1 together with average values. 

Reference device configuration with single NiO HTL shows a max (average) PCE of 14% (13.33%), while cells based on NiO/CuI HTL show a PCE that varies with the CuI concentration. The optimal configuration is found at 0.10 M concentration showing a max (average) PCE of 15.26% (14.23%) with an absolute PCE increase of +1.3% (0.90%), with respect to the PSC and the single NiO HTL. Improvement in a max and average V_OC_ was obtained for all double HTL cells concerning single NiO HTL (+0.04 V for 0.05 M CuI and +0.05 V for 0.10–0.20 M CuI). On the other hand, all PSCs with NiO /CuI HTL showed lower J_SC_ in comparison to the reference single HTL device. An average J_SC_ reduction of 6% and 16% was observed for PSCs with CuI concentration of 0.05 M and 0.20 M CuI, respectively, compared to the single NiO HTL, while for 0.10 M, the CuI concentration this reduction is limited to 1.7%. Fill Factor (FF) values are also improved for all devices with NiO/CuI HTL with respect to the single one (from 0.65 of the reference cell to 0.67–0.68 for the devices with CuI passivation). Calculated average shunt resistance (R_sh_) increased by increasing CuI concentration from a value of 1.5 kΩ·cm^2^ for the reference cell to 3.2 kΩ·cm^2^ for the device with 0.2 M CuI film. At the same time, by increasing the CuI concentration the average series resistance (R_S_) reduces: for 0.05 M CuI concentration R_S_ slightly reduces with respect to single NiO layer (~−3%), while at higher CuI concentrations (0.10–0.20 M) the R_S_ reduction is higher and of the order of ~−23%.

For comparison, we fabricated and characterized devices with single CuI HTLs. All the PV parameters reduce compared to single NiO and double NiO/CuI PSCs. The PCE of PSCs increased from a best 6.30% (average 4.41%) to 11.82 % (9.83%) by increasing the concentration of CuI form 0.05 M to 0.20 M, respectively. This trend is mainly correlated to the morphology of CuI film since a large quantity of pinholes significantly reduces R_sh_ to <1 kΩ·cm^2^ (versus > 1.5 kΩ·cm^2^ for reference NiO HTL device). Similarly, J_sc_, V_oc_, and FF increase by increasing the CuI concentration. 

Analyzing the effect of the double HTL onto the output characteristics and morphology of PSCs, we can point out that strong enhancement in R_SH_ is related to the capping of NiO micro-pinholes operated by the CuI layer. The reduction of shunts combined with a diminution of charge–recombination result in a Voc increase [51]. This effect can also be related to the chemical interaction of CuI at the NiO and perovskite interfaces providing bonds passivation with a consequent charge lifetime increase. A similar effect has been shown for silicon surface passivation obtained with iodine-containing compounds via dangling bonds saturation [52,53,54]. Ye et al. [55] studied CuI doped MAPbI_3_ film with extended X-ray absorption fine structure (EXAFS) analysis and found that CuI can interact with perovskite supplying iodine to fill vacancies and to influence the coordination of metal cations. Thus, CuI can effectively passivate the surface of perovskite film effectively reducing the trap states concentration. Moreover, copper atoms on surfaces of CuI particles are positive and coordinatively unsaturated that tends to interaction with defective perovskite structures [55]. At the same time, CuI does not induce chemical degradation at the interface with perovskite absorber films and shows promising stability results [56].

In order to estimate hysteresis effect, we measured forward and reverse JV curves for the best performing devices with single HTL configuration (NiO; CuI (0.05–0.20 M)) and NiO/CuI (0.10 M) at 23.5 mV/s scan rate. Results are presented in the Figure S2 (JV curves) and the Table S2 (calculated Hindex). For the reference PSCs with NiO HTL we obtained the absence of hysteresis, with a Hindex as low as 0.002. On the contrary, devices with single CuI HTLs, irrespective from CuI concentrations, present a large Hindex (0.288–0.3). Best performing device with double layer configuration NiO/CuI (0.10 M) show a small Hindex of 0.089. In principle, hysteresis in JV curves of PSCs are related to the charge accumulation induced by migration of ions and charged vacancies under electric field [57]. Double NiO/CuI (0.10 M) HTL configuration has much smaller H_index_ in comparison to single CuI HTLs, because NiO film effectively remove all leakages at hole collection interface. 

External quantum efficiency (EQE) measurements have been done for best-performing PSCs with NiO and NiO/CuI HTLs (CuI concentration 0.10 M), as shown in the EQE plot of Figure 4b. A reference device has a higher photon conversation level in the long wavelength region (505–740 nm) concerning double HTL, while the opposite is true for the short-wavelength region (325–425 nm). Reduction of EQE for NiO- CuI HTL with respect to the reference device in the long wavelength region (−4%) is related to the residual parasitic losses in transmission (see Figure S2 in ESI). On the contrary, the increase of the EQE in the short wavelength region (~+4%) is related to the positive impact of CuI to photon conversion. Such EQE spectrums with high conversation ratio in the short-wavelength (blue) range is typical for inverted PSC with a single CuI HTL [42], which can compensate losses typical for NiO in this region [58]. Short current densities extracted from the EQE spectra [59] show values of 19.58 mA/cm^2^ for the reference and 19.07 mA/cm^2^ for a device with the CuI interlayer, which fit well data extracted from J-V curves measured under 1 sun (see Table 1). We relate the decrease of Jsc to the parasitic absorption in wavelength region > 520 nm (as showed in Figure 3a) which clearly reduces the EQE (Figure 4b). 

**Figure 5:**
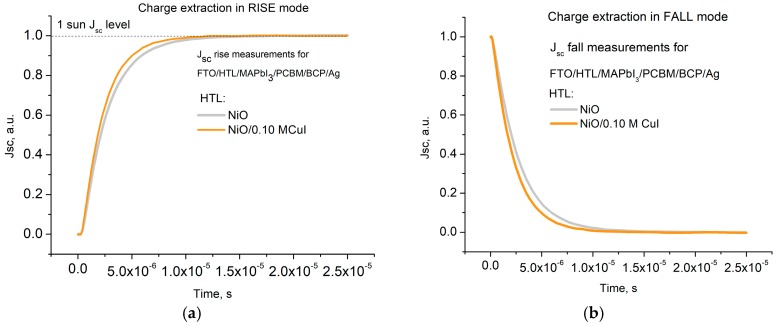
Transient photo current measurement s for the reference cell with single NiO HTL and double NiO/CuI (0.10 M) HTL in RISE (**a**) and FALL mode (**b**).

To investigate the charge transfer processes in PSCs with single and double HTLs, we performed transient photocurrent (TPC) and transient photovoltage (TPV) measurements (Figure 5 and Figure S4) [60]. In the measurement, the solar cell was switched ON to standard operating conditions (100 mW/cm^2^) and switched OFF to obtain transient processes of injection and relaxation marked as RISE and FALL modes, respectively. We measured transient characteristics for the reference device with NiO HTL and for PSC with NiO/CuI (0.10 M) HTL in both short and open circuit conditions. TPC. Figure 5a shows a faster RISE transient of the cell with NiO/CuI HTL with respect to one with single NiO HTL. This is a clear sign of the better hole injection dynamics for double HTL cell with respect to the single NiO HTL [61]. Similarly, FALL mode shows a faster relaxation dynamic in the device with double HTL to the single one. Both effects indicate that a reduction of interface traps between NiO and perovskite is achieved by inserting the CuI interlayer. The accelerated downturn of current density in FALL mode also points out that the interface has a reduced concentration of traps and parasitic charge accumulation which impede the flow of charges [61]. The observed charge dynamics correlate well to the trend of enhancement in PL quenching presented on Figure 3. TPV measurement (Figure S2a,b in E.S.I.) performed in RISE and FALL modes also confirms a faster collection of free charge carriers at passivated NiO HTL with a clear relaxation process (Figure S2b) rater faster in double HTL with respect to the single HTL, thus reaffirming the importance of CuI contribution to the reduction of trap states at the HTL/Perovskite interface.

**Figure 6:**
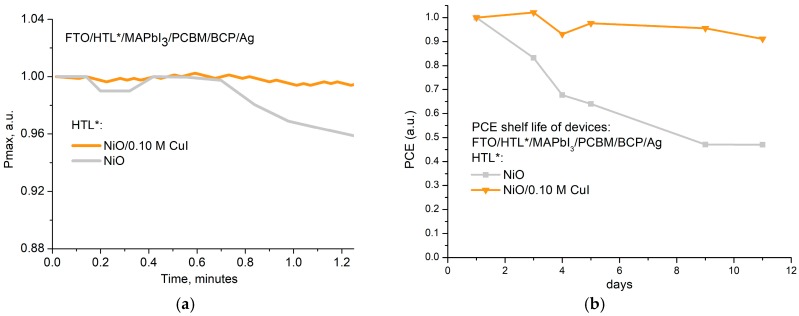
(**a**) Maximum power point tracking (MPPT) plot of devices with reference NiO HTL and CuI passivated double HTL, (**b**) PCE Shelf life measurements of devices with reference NiO HTL, and CuI passivated double HTL.

As we have seen in the previous section, the use of double NiO/CuI HTL strategy permit to increase charge transfer at the HTL/Perovskite interface and passivate the NiO surface. The latter, besides improving performance, also permits an improved stability of the cell. We measured an unencapsulated, best performing PSC with a single- and double-layer HTL, and with 1 Sun illumination that tracked the maximum power point (MPPT light soaking test, [62]) of an in shelf-life condition (ISOS-D-1 test). Results presented in Figure 6a,b demonstrate that both stress conditions had double NiO/CuI HTL and improved the stability of the PSC compared to the single HTL. 

MPPT data (Figure 6a) shows that the PSC with single NiO HTL starts to degrade the PCE already after 40 s of light soaking, while the PSC with double HTL does not degrade in the entire measurement range. Shelf-life data (Figure 6b) showed the same trend in an extended test period: after 12 days of shelf-life, the PCE of single HTL PSC reduces by more than 50% while the one of double HTL PSC reduces for less 10%.

Basically, the entire stability of both PSC depends on other factors such as Ag cathode diffusion and oxidation [63], MAPI decomposition [64] etc. [65], which are however kept equal in both devices.

### Figures, Tables and Schemes

## 4. Conclusions

In this work, we demonstrated a new solution processed method for passivation of NiO HTL in planar inverted perovskite solar cell with copper iodide. The incorporation of CuI interlayer at NiO–MAPbI_3_ heterojunction boundary mainly results in two observed effects:

(1) an increase of the efficiency of the solar cells from 14% for the devices with single NiO HTL to 15.2% to the devices with double NiO–CuI HTL;
(2) an enhancement of MPPT and shelf-life stability of PSCs with CuI passivation with respect to the reference.

It was revealed that device performance strongly correlates with the morphology of CuI film, which depends on the concentration used for the deposition. Improvements for the device performance were achieved with the use of 0.10 M concentration of CuI solution in acetonitrile, which allows forming 100 nm crystallites on the NiO surface after annealing at 100 °C. 

The increase of the PCE for the devices with CuI passivation was achieved due to the growth of V_oc_ (on average by +4.9%) and FF (+4.6%) caused by several factors. The presence of CuI interlayer on NiO surface in device structure reduced the density of pinholes, traps and significantly improved R_sh_ (from 1.6 to 3.3 kΩ·cm^2^ for passivation with 0.1 M solution). Moreover, developed double HTL structure showed lower series resistance (23% less) and provided better contact properties of hole collecting junction. 

The contribution of the CuI interlayer for charge transfer was investigated with TPC and TPV methods at 1 sun irradiation level. We observed that double NiO-CuI HTL configuration provides faster injection and relaxation processes in solar cell structures. The enhanced quenching of PL also confirmed improvement in hole injection for MAPbI_3_ film on a double HTL NiO-CuI as compared to single NiO. 

In this study, we demonstrated an effective approach for passivating of the NiO hole transport layer surface with simple deposition methods and cheap inorganic material (CuI), which improved the output characteristics as well as the stability of the devices. 

## Figures and Tables

**Table 1:** Photovoltaic parameters for the most efficient PSCs with the different HTLs used in work. PCE: power conversion efficiency; FF: fill factor; R_s_: average series resistance; R_sh_: average shunt resistance.

HTL Type		V_oc_, V	J_sc_, mA/cm^2^	FF	PCE, %	R_s_, Ω·cm^2^	R_sh_, kΩ·cm^2^
NiO	Best	1.02	−21.10	0.65	14.00	7.87	1.6
	Average *	1.01	−20.22	0.65 (±0.02)	13.33	7.88	1.5
		(±0.02)	(±0.50)		(±0.48)	(±0.24)	(±0.053)
CuI 0.05 M	Best	0.67	−14.25	0.66	6.30	4.75	0.34
	Average **	0.51	−13.83	0.62	4.41	4.81	0.29
		(±0.09)	(±0.51)	(±0.04)	(±1.14)	(±0.16)	(±0.02)
CuI 0.10 M	Best	0.73	−16.39	0.68	8.13	4.31	0.41
	Average **	0.68	−16.17	0.68	7.44	4.52	0.35
		(±0.07)	(±0.33)	(±0.02)	(±0.78)	(±0.13)	(±0.04)
CuI 0.20 M	Best	0.95	−17.57	0.71	11.82	4.77	0.66
	Average **	0.80	−17.07	0.70	9.83	4.80	0.61
		(±0.08)	(±0.38)	(±0.01)	(±1.06)	(±0.07)	(±0.02)
NiO/ 0.05 M CuI	Best	1.06	−18.48	0.68	13.32	7.61	2.4
	Average *	1.05	−19.02	0.67 (±0.02)	13.26	7.63	2.3
		(±0.01)	(±0.71)		(±0.37)	(±0.23)	(±0.081)
NiO/ 0.10 M CuI	Best	1.07	−20.60	0.69	15.26	6.05	3.3
	Average *	1.06	−19.88	0.68 (±0.02)	14.23	6.07	3.2
		(±0.02)	(±0.77)		(±0.72)	(±0.19)	(±0.112)
NiO/ 0.20 M CuI	Best	1.07	−17.20	0.68	12.51	6.06	3.9
	Average *	1.06	−17.40	0.64 (±0.02)	11.78	6.13	3.9
		(±0.03)	(±0.89)		(±0.64)	(±0.27)	(±0.135)

* ± spread of the data was calculated from the standard deviation for 16 devices for each HTL type. ** ± spread of the data was calculated from the standard deviation from 10 devices for each HTL type.

## Supplementary Materials

The following are available online at https://www.mdpi.com/1996-1944/12/9/1406/s1, Figure S1: SEM images of MAPbI3 films crystallized on the top of (a) NiO film; (b) NiO/CuI (0.10 M) film and (c) NiO/CuI (0.20 M), Figure S2: Statistical spread of output JV performance for fabricated devices, Figure S3: Hysteresis JV curves for the PSCs with different HTL types (a) NiO, (b) CuI (0.05 M), (c) CuI (0.10 M), (d) CuI (0.20 M) and (e) NiO/CuI (0.10 M), Figure S4: Transient photovoltage measurements for the reference cell with single NiO HTL and double NiO/CuI (0.10 M) HTL, Table S1: Thicknesses of films used in devices fabrication measured by stylus profilometer, Table S2: H_index_ calculated for PSCs with single HTL configurations and best performing NiO/CuI (0.10 M) double layer.
