# Supplementary material for: Copper Iodide Interlayer for Improved Charge Extraction and Stability of Inverted Perovskite Solar Cells
Source: Materials (Basel). 2019 Apr 30;12(9):1406. doi: 10.3390/ma12091406 (PMC6540312; doi:10.3390/ma12091406)
Supplement: Supplementary file 1 [file materials-12-01406-s001.pdf]

materials

Article

Copper Iodide Interlayer for Improved Charge Extraction and Stability of Inverted Perovskite Solar Cells

Danila Saranin [1], Pavel Gostischev [1], Dmitry Tatarinov [1], Inga Ermanova [1], Vsevolod Mazov [1], Dmitry Muratov [1], Alexey Tameev [2], Denis Kuznetsov [3], Sergey Didenko [4], and Aldo Di Carlo [1,5]*

1 L.A.S.E.—Laboratory for Advanced Solar Energy, National University of Science and Technology ''MISiS'', Leninskiy prospect 6, Moscow 119049, Russia; saranin.ds@misis.ru (D.S.), gostischev.pa@misis.ru (P.G.), tatarinov_dmitry1994@mail.ru (D.T.), ermanova.io@misis.ru (I.E.), mazov.v@misis.ru (V.M.), muratov@misis.ru (D.M.).
2 Laboratory ''Electronic and photon processes in polymer nanomaterials'', Russian Academy of Sciences A.N. Frumkin Institute of Physical chemistry and Electrochemistry, Leninskiy prospect 31k4, Moscow 119071, Russia; a.tameev@gmail.com
3 Department of Functional Nano Systems and High-Temperature Materials, National University of Science and Technology ''MISiS'', Leninskiy prospect 4, Moscow 119049, Russia; dk@misis.ru
4 Department of Semiconductor Electronics and Device Physics, National University of Science and Technology ''MISiS'', Krymskiy val 3, Moscow 119049, Russia; sdi13@mail.ru
5 CHOSE—Centre for Hybrid and Organic Solar Energy, Department of Electronic Engineering, University of Rome Tor Vergata, via del Politecnico 1, 00133 Rome, Italy
* Correspondence: aldo.dicarlo@uniroma2.it; Tel.: +39-320-439-18-61

Supplementary material for the paper:

Table S1. Thicknesses of films used in devices fabrication measured by stylus profilometer.

Layer	Thickness, nm
NiO	10 (±3.8)
CuI 0.05 M	23 (±19.2)
CuI 0.10 M	47 (±23.3)
CuI 0.20 M	74 (±32.1)
$MAPbI_3$	472 (±25.2)
PCBM	28 (±5.2)
BCP	8 (±2.1)
Ag	99 (±3.0)

We performed SEM imaging of perovskite films crystalized on NiO film and NiO/CuI stack with 0.10 M and 0.20 M concertation (Figure S1). Pin-hole free perovskite films with ~200–450 nm grain size was obtained without meaningful difference in morphology quality changes.

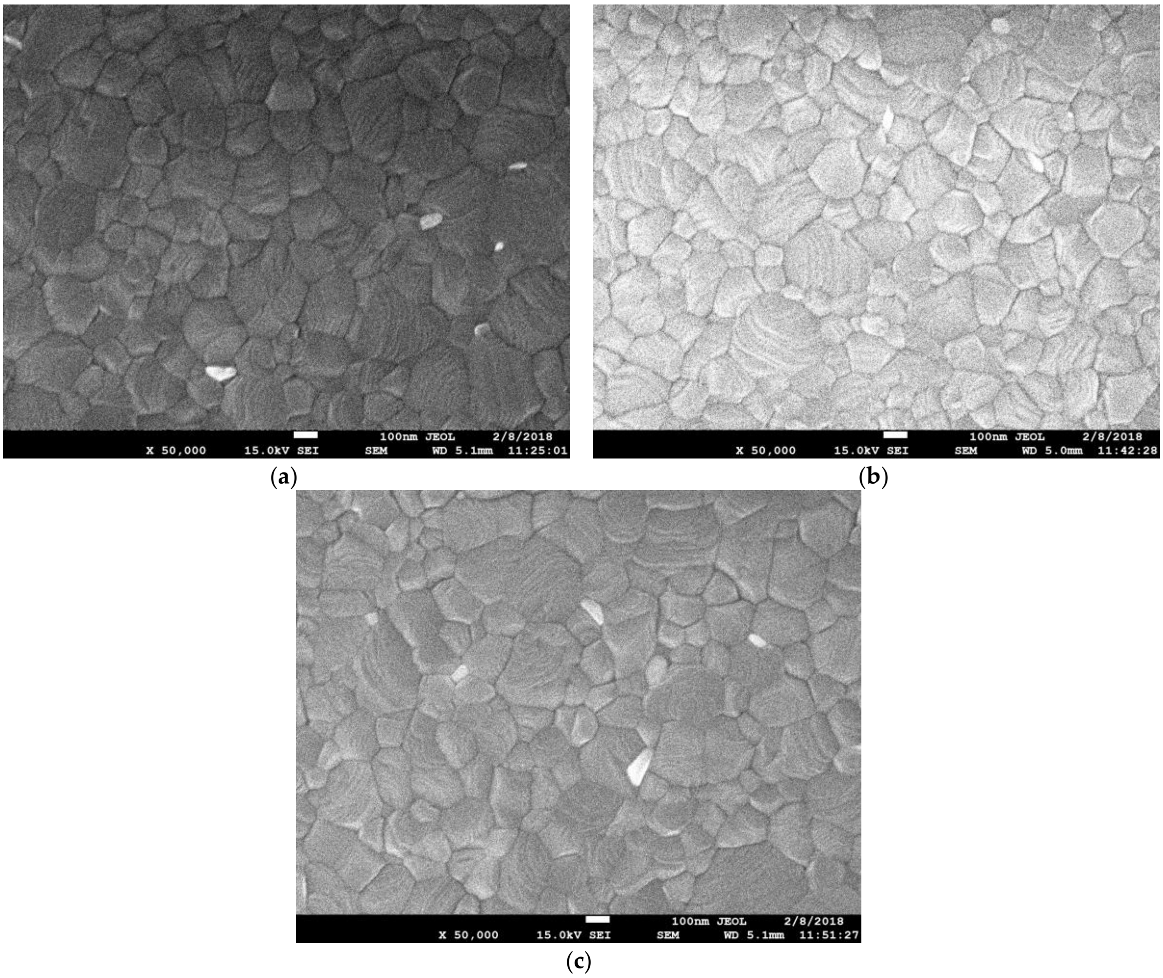

Figure S1. SEM images of $MAPbI_3$ films crystallized on the top of (**a**) NiO film; (**b**) NiO/CuI (0.10 M) film and (**c**) NiO/CuI (0.20 M).

Statistical spread of output JV performance for fabricated solar cells presented on Figure S1.

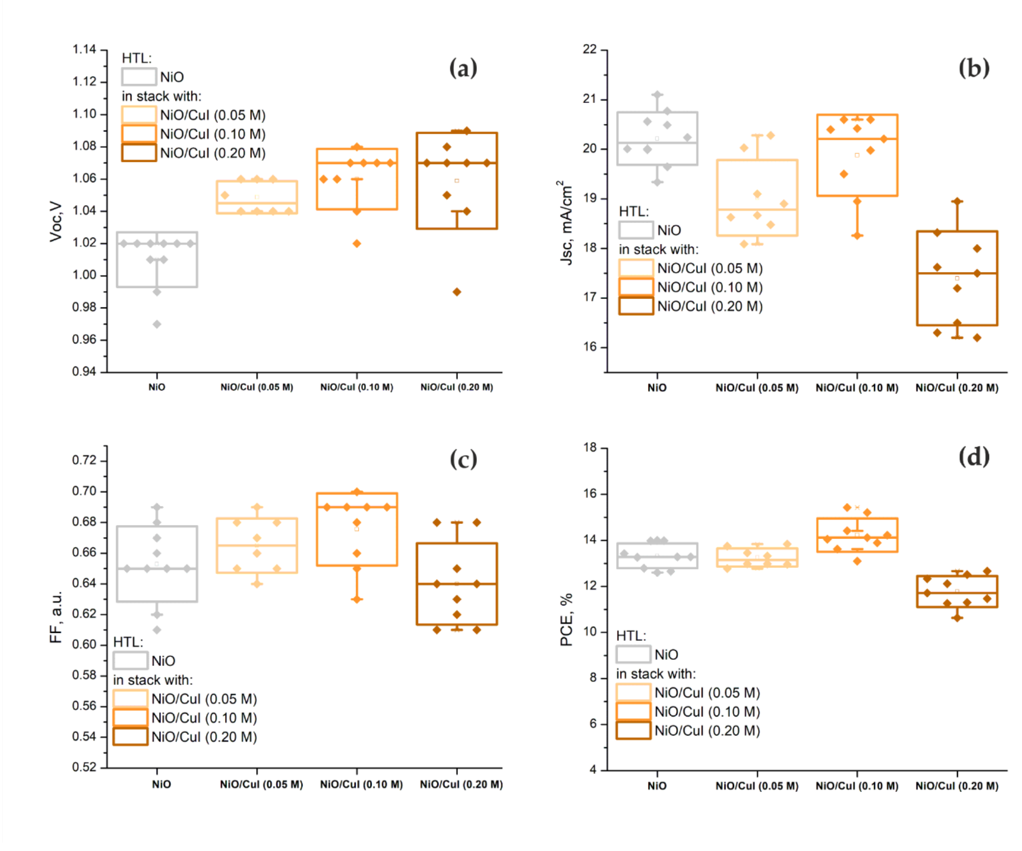

Figure S2. Statistical spread of output JV performance for fabricated devices (**a**) Voc range, (**b**) Jsc range, (**c**) FF range and (**d**) PCE range.

To quantitively compare the hysteresis effect we calculated H_{index} for JV curves measured at 23.5 mV/s scan rate (as for all devices) with Equation (S1):

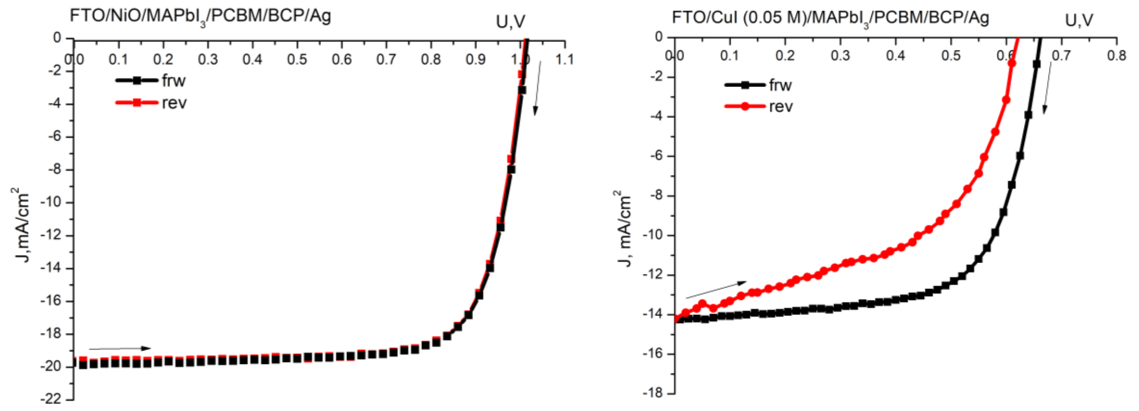

(**a**) (**b**)

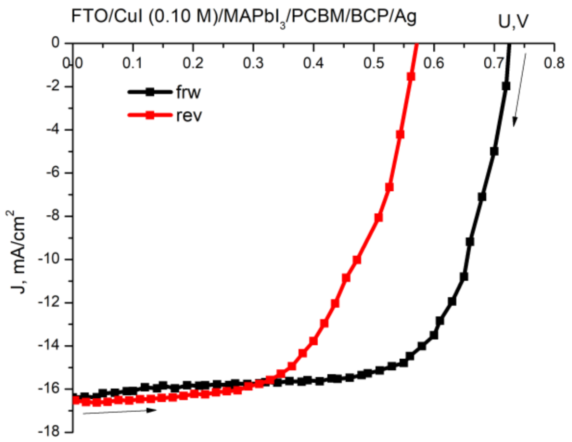

(**c**)

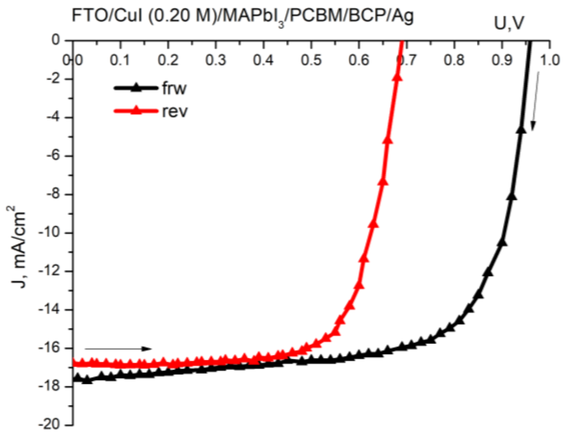

(**d**)

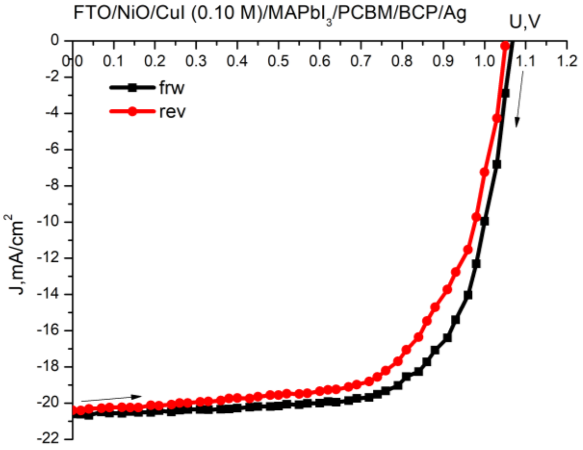

(**e**)

Figure S3. Hysteresis JV curves for the PSCs with different HTL types (**a**) NiO, (**b**) CuI (0.05 M), (**c**) CuI (0.10 M), (**d**) CuI (0.20 M) and (**e**) NiO/CuI (0.10 M).

To compare hysteresis effect quantitively by numbers we calculated H_{index} for JV curves measured at 23.5 mV/s scan rate (as for all devices) with Equation (S1):

$$H_{index} = \frac{PCE_{forward\ scan} - PCE_{reverse\ scan}}{PCE_{forward\ scan}} \quad (S1)$$

where PCE reverse scan - efficiency calculated from reverse scan of JV curve, %; PCE forward scan- efficiency calculated from forward scan of JV curve, %.

Table S2. H_{index} calculated for PSCs with single HTL configurations and best performing NiO/CuI (0.10 M) double layer.

HTL type	H_{index}, a.u.
NiO	0.002
CuI 0.05 M	0.288
CuI 0.10 M	0.322
CuI 0.20 M	0.294
NiO/CuI 0.10 M	0.089

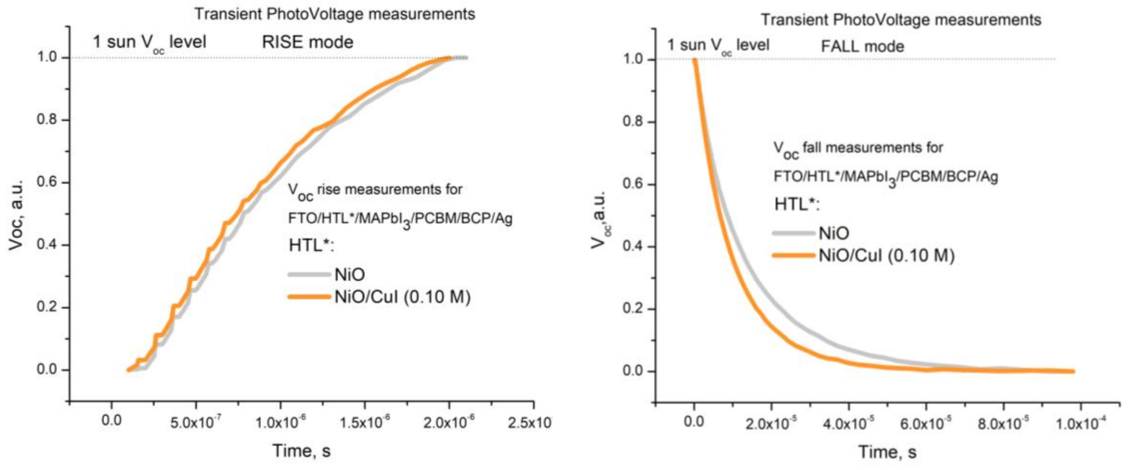

(**a**) (**b**)

Figure S4. Transient photo voltage measurements for the reference cell with single NiO HTL and double NiO/CuI (0.10 M) HTL in rise mode (**a**) and fall mode (**b**).
